# Network Pharmacology Analysis of the Therapeutic Potential of Colchicine in Acute Lung Injury

**DOI:** 10.1155/2024/9940182

**Published:** 2024-02-06

**Authors:** Fei Sun, Lijuan Zhang, Lulu Shen, Chunman Wang

**Affiliations:** ^1^Department of Anaesthesiology, Children's Hospital of Nanjing Medical University, No. 72 Guangzhou Road, Nanjing 210008, Jiangsu, China; ^2^Surgical Intensive Care Unit, Children's Hospital of Nanjing Medical University, No. 72 Guangzhou Road, Nanjing 210008, Jiangsu, China; ^3^Department of Anesthesiology, Huai'an Second People's Hospital and the Affiliated Huai'an Hospital of Xuzhou Medical University, No. 66 Huaihai South Road, Huai'an, Jiangsu, China; ^4^Pain Department, Hengshui People's Hospital, 180 People's East Road, Hengshui, Hebei, China

## Abstract

**Background:**

This study employed integrated network pharmacology approach to explore the mechanisms underlying the protective effect of colchicine against acute lung injury (ALI).

**Methods:**

We analyzed the expression profiles from 13 patients with sepsis-related ALI and 21 controls to identify differentially expressed genes and key modules. ALI-related genes were curated using databases such as DisGeNET, Therapeutic Target, and Comparative Toxicogenomics Database to curate ALI-related genes. Drug target fishing for colchicine was conducted using the DrugBank, BATMAN-TCM, STITCH, and SwissTargetPrediction. Potential drug-disease interactions were determined by intersecting ALI-associated genes with colchicine target genes. We performed comprehensive pathway and process enrichment analyses on these genes. A protein-protein interaction network was constructed, and topological analysis was executed. Additionally, an ALI mouse model was established to evaluate the effect of colchicine on CXCL12 and CXCR4 levels through western blot analysis.

**Results:**

Analysis revealed 23 potential colchicine-ALI interaction genes from the intersection of 253 ALI-associated genes and 389 colchicine targets. Functional enrichment analysis highlighted several inflammation-related pathways, such as cytokine-mediated signaling pathway, CXCR chemokine receptor binding, NF-kappa B signaling pathway, TNF signaling pathway, and IL-17 signaling pathway. The protein-protein interaction network demonstrated complex interactions for CXCL12 and CXCR4 among other candidate genes, with significant topological interaction degrees. *In vivo* studies showed that colchicine significantly reduced elevated CXCL12 and CXCR4 levels in ALI mice.

**Conclusion:**

Our findings suggest that colchicine's therapeutic effect on ALI might derive from its anti-inflammatory properties. Further research is needed to explore the specific mechanisms of colchicine's interaction with sepsis-induced ALI.

## 1. Introduction

Acute lung injury (ALI) represents a clinical syndrome arising from various lung injuries, which has a high incidence and causes substantial mortality [1]. Pneumonia, aspiration of acids, trauma, and sepsis are common causes of ALI [2]. ALI's pathology involves damage to both vascular endothelium and alveolar epithelium, marked by compromised alveolar-capillary membrane integrity, increased transepithelial neutrophil migration, and excessive proinflammatory cytokine release [3]. Besides supportive care, many potential pharmacologic treatments have been investigated and developed owing to a more in-depth understanding of the ALI pathophysiology [1, 4, 5]. Although the advances in medicine have delayed the disease's progress and prolonged the overall survival, an effective pharmacologic strategy for ALI remains elusive. Despite progresses in managing the disease and extending patient survival, ALI continues to be a primary cause of death among critically ill patients worldwide, representing a significant health burden. Therefore, more efforts are required to improve the progress further and reduce the mortality of ALI.

Colchicine is a classic and inexpensive anti-inflammatory drug of plant origin, which has been widely used to treat gout [6]. Its primary mechanism involves disrupting tubulin and inhibiting microtubule polymerization, leading to the downregulation of several inflammatory pathways and modulation of innate immunity [7]. Recent randomized controlled trials demonstrated colchicine's effectiveness in treating pericarditis and preventing atherosclerotic cardiovascular diseases [8], which inspired the further investigation of colchicine on other diseases.

The use of colchicine in mitigating lung injury in acute respiratory distress syndrome has recently garnered considerable interest [9–13]. It is reported that colchicine could improve ALI by reducing lung recruitment and activation of neutrophils [9]. Colchicine 1 mg/day significantly prolonged the survival rate for patients with severe COVID-19 combined ALI [10]. However, the specific mechanisms through which colchicine exerts its therapeutic effects on ALI are not fully understood. The bioinformatics tool provided a powerful method to analyze the mechanism underlying ALI and screen potential therapeutic drugs [14, 15].

In modern drug discovery and therapeutic design, the conventional “one target, one drug” approach of traditional pharmacology has transitioned to the more encompassing method of network pharmacology [16, 17]. This emerging field, merging systems biology with polypharmacology, probes the complex interplay between drugs and their myriad targets in expansive biological networks. Instead of a narrow focus on singular molecular interactions, network pharmacology highlights the interconnectedness of biological pathways, which are often disrupted in multifaceted diseases. By harnessing computational tools and vast biological datasets, this approach predicts potential drug targets, side effects, and synergistic combinations. Such a systems-centric viewpoint becomes indispensable for conditions like acute lung injury, where drugs, like colchicine, engage with several pathways, underscoring the necessity of viewing their impacts within a wider biological framework.

In our study, we employed comprehensive bioinformatics methodologies to elucidate the underlying mechanisms of colchicine's protective effect against ALI.

## 2. Methods

### 2.1. Differential Gene Expression

We analyzed gene expression profiles from GSE10474, comprising whole blood samples collected within 48 hours of admission from 34 patients. This group included 13 patients with sepsis-related ALI and 21 with sepsis alone. ALI was defined according to the American-European Consensus Conference on acute respiratory distress syndrome [18]. Blood samples were stored in the PAXgene Blood RNA Tubes at −70°C for RNA preservation. The array expression profiles were examined using the Affymetrix Human Genome U133A 2.0 Array GPL571. Detailed methodology is described elsewhere [19]. After background correction and quantile normalization on the expression profiles, the probes results were annotated to gene symbols based on the platform annotation files. We calculated the average expression level for probes matching to the same gene for the following analysis. Differentially expressed genes (DEGs) were identified using a threshold of *P* value < 0.05 and an absolute log2 fold-change ≥1.

### 2.2. Weighted Gene Coexpression Network Analysis

Weighted gene coexpression network analysis (WGCNA) serves as a systems bioinformatics approach to examine correlation patterns of genes across microarray samples and identify the highly correlated modules [20, 21]. The WGCNA R software package has been widely applied to construct coexpression networks, detect modules, identify correlated genes, evaluate module relationships, and visualize [21].

In this study, we utilized the one-step network construction method to analyze the expression profiles from GSE10474, reconstructing gene coexpression networks using the WGCNA package. Adhering to the scale-free topology criterion, we established a soft threshold power of 4 (*β* = 4, with an *R*-square of 0.89) for creating the weighted adjacency matrix. Subsequently, the module eigengene, quantified by the first principal component, was used to evaluate module similarity and assess relationships between the module and specific traits. We then determined the correlation strength based on module significance, enabling the identification of key modules in the study.

### 2.3. Target Fishing Based on Digital Platforms

A comprehensive strategy was used to fish potential drug-disease interaction targets based on multiple discovery platforms, including DisGeNET [22], Therapeutic Target Database [23], Comparative Toxicogenomics Database [24], DrugBank, BATMAN-TCM tool, STITCH, and SwissTargetPrediction.

The ALI-related targets were fished from the DisGeNET [22], Therapeutic Target Database [23], and Comparative Toxicogenomics Database [24]. DisGeNET integrates data from expert-curated repositories, animal models, genome-wide association studies, and scientific literature [22]. The Therapeutic Target Database collected known/explored therapeutic targets, the targeted diseases, and the corresponding target-directed drugs [23]. Successful and clinical trial targets were included in the Therapeutic Target Database. The Comparative Toxicogenomics Database compiles both curated and inferred gene-disease relationships [24]. In the Comparative Toxicogenomics Database, the curated gene-disease relationships are acquired from the OMIM database or published literature, while the inferred relationships are established based on curated chemical-gene interactions. It should be noted that only curated disease-related genes and inferred genes with high confidence (defined as an inference score above 100) from the Comparative Toxicogenomics Database were used in this study. Then, DEGs, genes in key modules, and database-screened genes were identified as ALI-related genes.

Moreover, we conducted drug target fishing using four databases: DrugBank [25], BATMAN-TCM (Bioinformatics Analysis Tool for Molecular mechANism of Traditional Chinese Medicine) [26], STITCH [27], and SwissTargetPrediction [28]. DrugBank is a knowledgebase combining detailed drug information on comprehensive drug action and drug targets [25]. BATMAN-TCM tool is a specialized online bioinformatics platform designed to predict potential targets of traditional Chinese herb compounds [26]. STITCH is another integrated database for effective *in silico* drug discovery [27]. SwissTargetPrediction provided an easy-to-use website tool to estimate the possible targets of a small molecule [28]. The verified and predictive colchicine target genes were harvested using the keyword of “colchicine” or its canonical SMILES (CC(=O)NC1CCC2=CC(=C(C(=C2C3=CC=C(C(=O)C=C13)OC)OC)OC)OC) from the four drug target databases. Finally, the ALI-related genes and colchicine target genes were overlapped to acquire candidate drug-disease interaction genes.

### 2.4. Functional Enrichment Analysis on the Colchicine-ALI Interaction Genes

Enrichment analyses in our study were performed using the clusterProfiler R software package [29]. The Gene Ontology (GO) enrichment analysis is to elucidate the biological processes, cellular components, and molecular functions. Then, the Kyoto Encyclopedia of Genes and Genomes (KEGG) enrichment was performed to identify the related pathway. We also utilized Metascape, a web-based tool designed for experimental biologists to perform comprehensive analyses and interpretations of gene clusters [30]. The Metascape tool was used to perform pathway and process enrichment analysis based on KEGG pathway, GO biological processes, Reactome gene sets, canonical pathways, Corum, and WikiPathways.

### 2.5. Construction of Protein-Protein Interaction Network and Topological Analysis

STRING database, which encompasses a comprehensive collection of known and predicted protein-protein interactions (PPIs), both direct (physical) and indirect (functional), was utilized for network pharmacology analysis [31]. We input the candidate drug-disease interaction genes into the STRING database, extracting interactions with a medium confidence cutoff score of 0.4. The resulting PPI network was then constructed and visualized using Cytoscape software (version 3.9.1) [32].

For the analysis of topological parameters, we employed the network analyzer, a plug-in of Cytoscape, to evaluate the mean and maximum degrees of nodes within the PPI network. Molecular Complex Detection (MCODE) algorithm (Version 1.6) [33], adept at identifying densely connected regions in large PPI networks, was applied. The settings for this analysis included a degree cutoff of 2, a node score cutoff of 0.2, and a K-core of 2, enabling the identification of highly interacting genes within the network.

### 2.6. Animal Validation

All procedures involving mice in this study adhered to NIH guide of humane use and care of animals and were approved by institutional approved protocols. Female C57BL/6 J mice, aged 6–8 weeks, were acquired from the Model Animal Research Center of Nanjing University. Throughout the experiment, mice were housed in a controlled environment with a 12-hour light/dark cycle and had free access to food and water. The mice were randomized into three groups: ALI group, colchicine treatment group (ALI with colchicine treatment), and control group. Group sizes were determined based on previous research [34, 35]. To establish the ALI model, mice were exposed to 0.8 mg/ml nebulized lipopolysaccharide (*Escherichia coli* O111: B4, Sigma, USA) diluted in 0.9% NaCl for 30 minutes, consistent with previous studies [36, 37]. The control group received only nebulized 0.9% NaCl. In the colchicine treatment group, colchicine (Sigma, USA) was administrated at a dose of 0.1 mg/kg at 8 h and 12 h after lipopolysaccharide treatment. All mice were maintained under identical conditions and received the same anesthesia. At 48 hours after lipopolysaccharide or NaCl treatment, mice were intraperitoneally given overdose pentobarbital to perform euthanasia, and lung samples were collected. Lung tissue proteins (20 *µ*g per lane) were separated on 15% SDS-PAGE gels (Beyotime, China) and transferred onto polyvinylidene difluoride (PVDF) membranes (Amersham Biosciences, USA). Membranes were blocked by 5% nonfat milk in TBS-Tween 20 (0.1% TBST) at room temperature for 1 h, then incubated overnight at 4°C with primary antibodies for CXCL12 (ab155090, Abcam, USA), CXCR4 (ab124824, Abcam, USA), and GAPDH (#2118, Cell Signaling Technology, USA). Horseradish peroxidase-conjugated anti-rabbit secondary antibodies (1 : 10000) were then applied for 1 hour at room temperature. Visualization was done using the SuperSignal West Pico Chemiluminescence ECL Kit (Pierce), and band densities were quantified using ImageJ software (National Institutes of Health, Bethesda, USA), normalized to control treatment and GAPDH. The experimental procedure followed the Guidelines for the Care and Use of Laboratory Animals, with ethical approval from the Ethics Committee of Xuzhou Medical University.

### 2.7. Statistical Analysis

Differential expression analysis in our study was conducted using the *t*-test, applied through the limma R software package (version 3.52.4) [38]. In the animal study, the one-way ANOVA analysis was applied to compare the relative protein levels. All statistical analyses and visualizations were performed using R software (version 4.1, R Core Team) and Prism software 8.0 (GraphPad, San Diego, USA). A *P* value of less than 0.05 was considered statistically significant in our analysis.

## 3. Results

### 3.1. Differentially Expressed Genes and Key Modules

**Figure 1:**
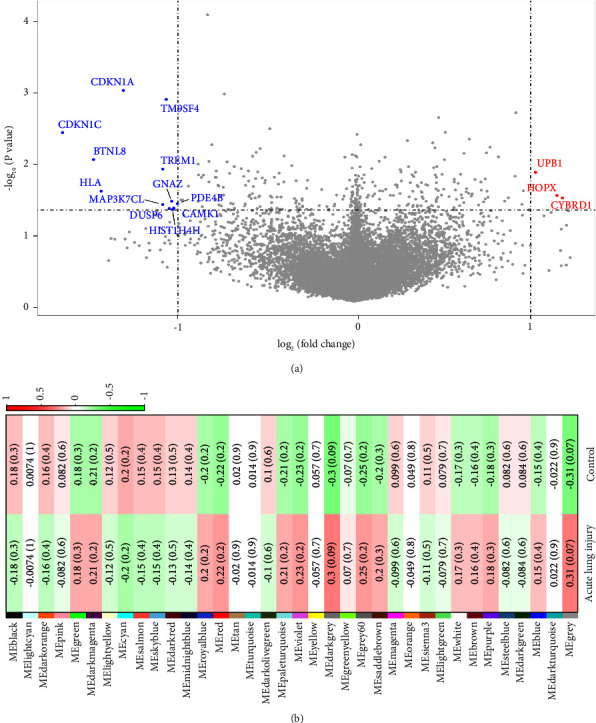
Bioinformatics analysis on expression profiles comparing patients with ALI to those without ALI. (a) Volcano plots of the differential gene expressions. Significantly upregulated genes are indicated by red dots, while significantly downregulated genes are represented by blue dots. The criteria for identifying differentially expressed genes were set at a *P* value < 0.05 and an absolute log2 fold-change ≥1. (b) Association between the gene module and the prevalence of acute lung injury. Each cell is annotated with the correlation coefficient and corresponding *P* value, indicating the relationship of each module with the clinical trait. The cell color from green to red represents the increasing correlation coefficient from −1 to 1 (legend at right). No significant modules were observed (all *P* > 0.05). The sample sizes were 13 in the ALI group and 21 in the control group.

Our analysis identified a total of 15 DEGs when comparing patients with ALI to those without ALI. Three genes were significantly upregulated (*UPB1*, *HOPX*, and *CYBRD1*), and 12 were significantly downregulated (*CDKN1A*, *TM9SF4*, *CDKN1C*, *BTNL8*, *TREM1*, *HLA*, *GNAZ*, *PDE4B*, *MAP3K7CL*, *CAMK1*, *DUSP6*, and *HIST1H4H*) between the two groups (Figure 1(a)). The expression fold change and the *P* values of DEGs are provided in Supplementary Table 1.

WGCNA algorithm was employed to construct a coexpression network to investigate biologically meaningful gene clusters. A total of 35 modules were identified, and the genes not assigned to any modules were categorized into the grey module. The module eigengenes of each module were developed, and the association between module eigengene and the prevalence of ALI were calculated. Each module was randomly assigned a distinct color for better data visualization. We observed that all modules showed a weak or moderate association with ALI, and there was no significant module (all *P* values > 0.05; Figure 1(b)).

### 3.2. Candidate Drug-Disease Interaction Genes

**Figure 2:**
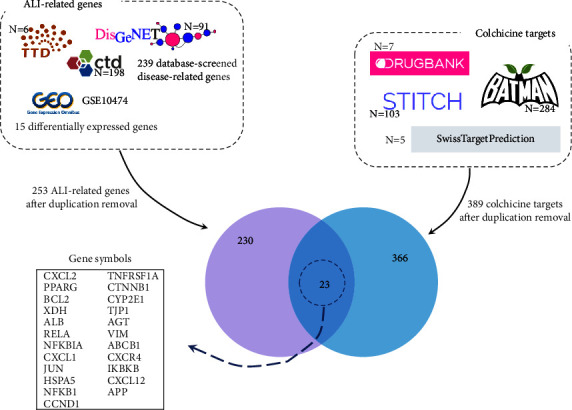
The flowchart to identify the colchicine-ALI interaction genes. The number attached to the database name refers to the number of genes acquired from the database. After duplicated removal, 253 ALI-related genes were acquired from the DisGeNET, Therapeutic Target Database, Comparative Toxicogenomics Database, and GSE10474. 389 verified or predictive colchicine targets were identified based on the DrugBank, BATMAN-TCM, STITCH, and SwissTargetPrediction. Finally, a total of 23 potential colchicine-ALI interaction genes were acquired. ALI: acute lung injury.

Figure 2 summarizes a flowchart to identify the candidate drug-disease interaction genes. 91 disease-related genes were obtained from the DisGeNET, six from the Therapeutic Target Database, and 198 from the Comparative Toxicogenomics Database. After eliminating duplicates, 239 database-screened disease-related genes remained. These database-screened disease-related genes were then merged with the DEGs, and a total of 253 genes were identified as ALI-related ones after duplicate removal. For colchicine targets, 7, 284, 103, and 102 candidate colchicine targets were acquired from the DrugBank, BATMAN-TCM, STITCH, and SwissTargetPrediction, respectively. After deleting the duplicated genes, 389 verified or predictive colchicine targets were acquired.

Finally, the 253 ALI-related genes and the 389 colchicine targets were overlapped, and a total of 23 potential colchicine-ALI interaction genes were acquired, including *CXCL2*, *PPARG*, *BCL2*, *XDH*, *ALB*, *RELA*, *NFKBIA*, *CXCL1*, *JUN*, *HSPA5*, *NFKB1*, *CCND1*, *TNFRSF1A*, *CTNNB1*, *CYP2E1*, *TJP1*, *AGT*, *VIM*, *ABCB1*, *CXCR4*, *IKBKB*, *CXCL12*, and *APP*.

### 3.3. Functional Enrichment Analysis

**Figure 3:**
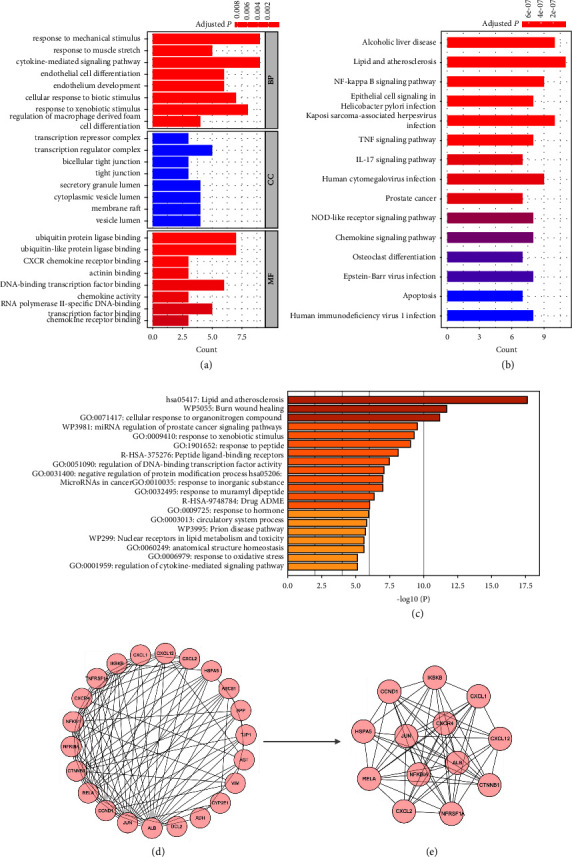
Functional enrichment analysis and PPI network. The functional enrichment analysis on the colchicine-ALI interaction genes based on (a) Gene Ontology and (b) Kyoto Encyclopedia of Genes and Genomes databases. (c) Comprehensive pathway and process enrichment analysis based on Kyoto Encyclopedia of Genes and Genomes pathway, Gene Ontology biological processes, Reactome gene sets, canonical pathways, Corum, and WikiPathways. (d) The PPI network of the colchicine-ALI interaction genes. (e) The cluster subnetwork based on the MCODE algorithm. PPI: protein-protein interaction.

Functional enrichment analysis was conducted on these identified drug-disease interaction genes. This analysis utilized the GO and KEGG databases to provide insights into the biological processes, cellular components, molecular functions, and pathways associated with these genes. The results of the GO enrichment analysis are summarized in Figure 3(a). The enriched biological processes included response to mechanical stimulus, response to muscle stretch, cytokine-mediated signaling pathway, endothelial cell differentiation, and endothelium development. The enriched cell component included transcription repressor complex, transcription regulator complex, bicellular tight junction, tight junction, and secretory granule lumen. The enriched molecular function included ubiquitin protein ligase binding, ubiquitin-like protein ligase binding, CXCR chemokine receptor binding, actinin binding, and DNA-binding transcription factor binding. Moreover, several inflammation-related pathways were enriched in the KEGG pathway analysis, including NF-kappa B signaling pathway, TNF signaling pathway, and IL-17 signaling pathway (Figure 3(b)). Additionally, Figure 3(c) lists the top 15 clusters with enriched representative terms, such as response to oxidative stress, regulation of cytokine-mediated signaling pathway, and nuclear receptors in lipid metabolism and toxicity.

### 3.4. PPI Network Based on the Colchicine-ALI Interaction Genes

The 23 colchicine-ALI interaction genes were imported into the STRING database. This process resulted in a PPI network comprising 23 nodes and 121 edges, with an average node degree of 10.5. The PPI network is displayed in Figure 3(d). One cluster subnetwork of 13 genes was created based on the MCODE algorithm, which included *CXCR4*, *CXCL1*, *CXCL2*, *CXCL12*, *CCND1*, *CTNNB1*, *IKBKB*, *RELA*, *JUN*, *HSPA5*, *ALB*, *NFKBIA*, and *TNFRSF1A* (Figure 3(d)). Importantly, a complex interaction of CXCR4 and CXCL12 with other candidate genes was observed, which shows topological high interaction degrees.

### 3.5. Colchicine Alleviates Increased Levels of CXCL12 and CXCR4 in ALI

**Figure 4:**
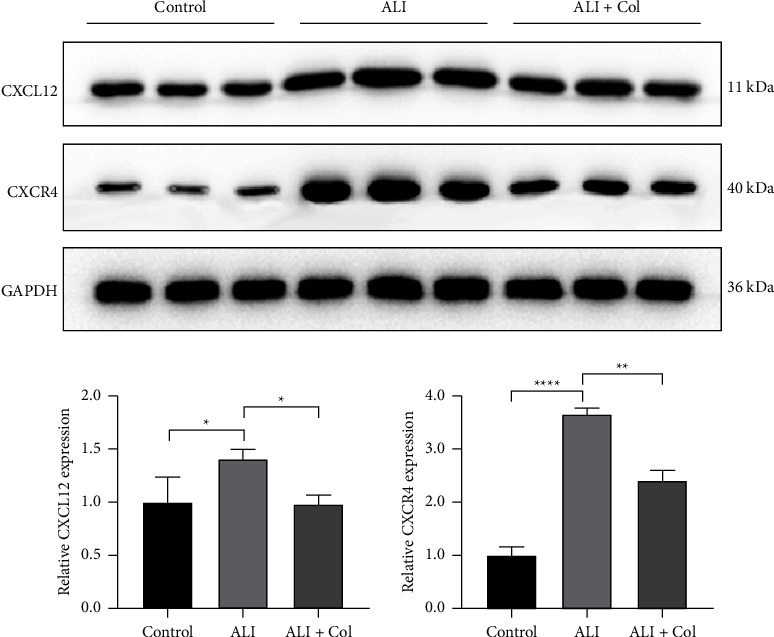
The protein expression of CXCL12 and CXCR4 in ALI mice by western blot. *N* = 3 in each group. ALI: acute lung injury; Col: colchicine. ^*∗*^ < 0.01, ^*∗∗*^ < 0.001, and ^*∗∗∗∗*^ < 0.0001.

Western blot revealed that the expression of CXCL12 and CXCR4 was significantly higher in the ALI mice compared to the control group (Figure 4). However, after colchicine administration, there was a significant reduction in the expression of both CXCL12 and CXCR4 in the treated mice, compared to those in the ALI group without colchicine treatment.

## 4. Discussion

Colchicine is an anti-inflammatory medication, notable for its potent antioxidant and antiapoptotic effects [7, 39]. Colchicine has been considered the first-line treatment for autoinflammatory syndromes (e.g., acute gouty arthritis and recurrent pericarditis). This preference is due to its ability to inhibit neutrophil adhesion and mobilization [40]. Recent studies suggested that colchicine is potentially used in many other diseases, such as myocardial infarction injury [41, 42], periodontitis [43], and pulmonary arterial hypertension [44]. Considering the well-recognized anti-inflammatory property, applying colchicine to treat ALI has achieved wide attention. Our study identified 23 candidate target genes of colchicine against ALI. The functional enrichment analysis suggests that the colchicine-ALI interaction genes are involved in several inflammation-related pathways, such as NF-kappa B signaling pathway, TNF signaling pathway, and IL-17 signaling pathway. This *in silico* study provides preliminary evidence that colchicine's therapeutic effect may primarily stem from its anti-inflammatory effect.

ALI is a life-threatening syndrome characterized by infiltrated neutrophils and leucocytes and increased inflammation levels in pulmonary tissue [45, 46]. Previous studies have demonstrated that the activation of CXCL12/CXCR4 signaling pathway could contribute the neutrophil accumulation and retention in the lungs during ALI [47] and accelerate lung fibrosis [48]. Based on the PPI network and the MCODE analysis, our study revealed the close interaction of CXCL12 and CXCR4 with other candidate genes. CXCR4 is a G-protein-coupled chemokine receptor with 7 transmembrane domains on the cell surface [49]. CXCL12 (also termed SDF-1) is classified as a homeostatic chemokine, which transduces signals via increasing intracellular calcium ion levels. The chemokine CXCL12 could interact with its receptor, CXCR4, forming a coupled molecular pair often referred to as a chemotaxis axis. This interaction constitutes a significant signal transduction pathway in the body, playing a crucial role in various biological processes. Our results suggested that CXCL12/CXCR4 pathway might be an important pathway bridging the therapeutic effect of colchicine against ALI. The following studies should further investigate the role of the CXCL12/CXCR4 axis in the interplay between colchicine and ALI.

Many animal studies have investigated the application of colchicine to treat ALI [9–13]. Zhang et al. [11] explored the therapeutic effect of colchicine against severe acute pancreatitis-induced ALI in a rat model. Pretreatment with colchicine could significantly mitigate ALI by reducing lung tissue cell apoptosis, alleviating inflammatory responses, and attenuating the NF-*κ*B, STAT3, and AKT signaling in ALI rats [11]. Increased expression of Nrf2 and HO-1 was also observed in lung tissues after colchicine treatment, which indicated limited oxidative stress. In another animal study by Yue et al. [12], the therapeutic effect of colchicine was investigated in LPS-induced ALI rats. After colchicine treatment, pulmonary tissue damage of ALI was significantly attenuated via inhibiting JNK, Erk1/2, and P-38 activation [12]. Similarly, a recent study also reported the therapeutic effect of colchicine on oleic acid-induced acute respiratory distress syndrome rats [9]. The colchicine group showed substantially reduced histological by 61% compared to the sham group and improved PaO_2_/FiO_2_ from 66 to 246. Interestingly, MPO immunostaining suggested that colchicine treatment could reduce the neutrophil recruitment in the lung but not reduce the circulating neutrophilia level [9].

Importantly, colchicine has recently been evaluated as a treatment for patients with COVID-19 pneumonia and acute respiratory distress syndrome [10]. In a case-control study comprising 122 patients, those administered 1 mg/day of colchicine, along with the standard-of-care treatment (hydroxychloroquine and/or intravenous dexamethasone and/or lopinavir/ritonavir) were compared with 140 patients who received only the standard-of-care treatment. After a follow-up of 21 days, patients treated with colchicine exhibited a notably improved survival (84.2% vs 63.6%, *P*=0.001), with a HR of 0.151 (95% confidence interval = 0.062–0.368, *P* < 0.0001) [10]. In addition, a clinical trial on colchicine to treat post-COVID-19 pulmonary fibrosis was also underway (NCT04818489). Consistent with previous studies, we provided *in silico* evidence to support colchicine against ALI and investigated the possible underlying mechanisms.

Furthermore, some limitations of this study should be mentioned. First, the blood samples of GSE10474 were obtained from sepsis patients with or without ALI in intensive care. These sepsis patients were usually with other diseases which might interfere with the results of genetic analysis. Second, colchicine's therapeutic index is relatively low, with effective steady-state plasma concentrations ranging between 0.5 and 3 ng/ml [50]. Given its narrow safety margin, colchicine usage in elderly patients with renal insufficiency and heart disease requires careful consideration. However, this study did not provide the dose-dependent effect of colchicine on ALI. Third, while our findings suggest that colchicine's therapeutic impact on ALI may be attributed to its anti-inflammatory properties and possibly involves the CXCL12/CXCR4 axis, we did not perform external validation. The following validation based on animal models would be necessary. Therefore, our results should be taken with caution, and the side effects of colchicine should be considered. Forth, we acknowledged that using only one dataset (GSE10474) is limited in size. Still, our research focuses on analyzing the specific dataset GSE10474, which provides valuable insights into the topic of sepsis-induced AKI. Despite being limited in size, these results still add valuable information to the existing literature.

## 5. Conclusion

This study suggested that colchicine's therapeutic efficacy in treating ALI might stem from its anti-inflammatory effect and involve the CXCL12/CXCR4 axis. Further validation is required to investigate the interaction mechanisms between colchicine and sepsis-induced ALI.

## Data Availability

The data were obtained from the GEO database (https://www.ncbi.nlm.nih.gov/geo/query/acc.cgi?acc=gse10474).

## Supplementary Materials

Supplementary Materials Supplementary Table 1. The expression fold change and the *P* values of the 15 differentially expressed genes.

## References

[1] Johnson E. R., Matthay M. A. (2010). Acute lung injury: epidemiology, pathogenesis, and treatment. *Journal of Aerosol Medicine and Pulmonary Drug Delivery*.

[2] Zhong W., Cui Y., Yu Q. (2014). Modulation of LPS-stimulated pulmonary inflammation by Borneol in murine acute lung injury model. *Inflammation*.

[3] Matthay M. A., Zimmerman G. A. (2005). Acute lung injury and the acute respiratory distress syndrome: four decades of inquiry into pathogenesis and rational management. *American Journal of Respiratory Cell and Molecular Biology*.

[4] Morris P. E., Papadakos P., Russell J. A. (2008). A double-blind placebo-controlled study to evaluate the safety and efficacy of L-2-oxothiazolidine-4-carboxylic acid in the treatment of patients with acute respiratory distress syndrome. *Critical Care Medicine*.

[5] Liu K. D., Levitt J., Zhuo H. (2008). Randomized clinical trial of activated protein C for the treatment of acute lung injury. *American Journal of Respiratory and Critical Care Medicine*.

[6] McKenzie B. J., Wechalekar M. D., Johnston R. V., Schlesinger N., Buchbinder R. (2021). Colchicine for acute gout. *Cochrane Database of Systematic Reviews*.

[7] Leung Y. Y., Yao Hui L. L., Kraus V. B. (2015). Colchicine--Update on mechanisms of action and therapeutic uses. *Seminars in Arthritis and Rheumatism*.

[8] Tardif J. C., Kouz S., Waters D. D. (2019). Efficacy and safety of low-dose colchicine after myocardial infarction. *New England Journal of Medicine*.

[9] Dupuis J., Sirois M. G., Rheaume E. (2020). Colchicine reduces lung injury in experimental acute respiratory distress syndrome. *PLoS One*.

[10] Scarsi M., Piantoni S., Colombo E. (2020). Association between treatment with colchicine and improved survival in a single-centre cohort of adult hospitalised patients with COVID-19 pneumonia and acute respiratory distress syndrome. *Annals of the Rheumatic Diseases*.

[11] Zhang D., Li L., Li J. (2022). Colchicine improves severe acute pancreatitis-induced acute lung injury by suppressing inflammation, apoptosis and oxidative stress in rats. *Biomedicine & Pharmacotherapy*.

[12] Yue Q., Liu T., Cheng Z. (2020). Protective effect of colchicine on LPS-induced lung injury in rats via inhibition of P-38, ERK1/2, and JNK activation. *Pharmacology*.

[13] Al-Kuraishy H. M., Hussien N. R., Al-Niemi M. S., Al-Gareeb A. I. (2021). Colchicine in the management of Covid-19: with or lieu of evidence. *Journal of Pakistan Medical Association*.

[14] Mao K., Geng W., Liao Y. (2020). Identification of robust genetic signatures associated with lipopolysaccharide-induced acute lung injury onset and astaxanthin therapeutic effects by integrative analysis of RNA sequencing data and GEO datasets. *Aging (Albany NY)*.

[15] Cao F., Wang C., Long D., Deng Y., Mao K., Zhong H. (2021). Network-based integrated analysis of transcriptomic studies in dissecting gene signatures for LPS-induced acute lung injury. *Inflammation*.

[16] Hopkins A. L. (2008). Network pharmacology: the next paradigm in drug discovery. *Nature Chemical Biology*.

[17] Eurekaselect (2023). Pharmacogenetics in Personalized Medicine: underlying mechanisms and practical applications. https://eurekaselect.com/call-for-papers-detail/4907/specialissue.

[18] Bernard G. R., Artigas A., Brigham K. L. (1994). The American-European Consensus Conference on ARDS. Definitions, mechanisms, relevant outcomes, and clinical trial coordination. *American Journal of Respiratory and Critical Care Medicine*.

[19] Howrylak J. A., Dolinay T., Lucht L. (2009). Discovery of the gene signature for acute lung injury in patients with sepsis. *Physiological Genomics*.

[20] Zhang B., Horvath S. (2005). A general framework for weighted gene co-expression network analysis. *Statistical Applications in Genetics and Molecular Biology*.

[21] Langfelder P., Horvath S. (2008). WGCNA: an R package for weighted correlation network analysis. *BMC Bioinformatics*.

[22] Pinero J., Sauch J., Sanz F., Furlong L. I. (2021). The DisGeNET cytoscape app: exploring and visualizing disease genomics data. *Computational and Structural Biotechnology Journal*.

[23] Zhou Y., Zhang Y., Lian X. (2022). Therapeutic target database update 2022: facilitating drug discovery with enriched comparative data of targeted agents. *Nucleic Acids Research*.

[24] Grondin C. J., Davis A. P., Wiegers J. A. (2021). Predicting molecular mechanisms, pathways, and health outcomes induced by Juul e-cigarette aerosol chemicals using the Comparative Toxicogenomics Database. *Current Research in Toxicology*.

[25] Wishart D. S., Knox C., Guo A. C. (2008). DrugBank: a knowledgebase for drugs, drug actions and drug targets. *Nucleic Acids Research*.

[26] Liu Z., Guo F., Wang Y. (2016). BATMAN-TCM: a bioinformatics analysis tool for molecular mechANism of traditional Chinese medicine. *Scientific Reports*.

[27] Szklarczyk D., Santos A., von Mering C., Jensen L. J., Bork P., Kuhn M. (2016). Stitch 5: augmenting protein-chemical interaction networks with tissue and affinity data. *Nucleic Acids Research*.

[28] Daina A., Michielin O., Zoete V. (2019). SwissTargetPrediction: updated data and new features for efficient prediction of protein targets of small molecules. *Nucleic Acids Research*.

[29] Wu T., Hu E., Xu S. (2021). clusterProfiler 4.0: a universal enrichment tool for interpreting omics data. *Innovation*.

[30] Zhou Y., Zhou B., Pache L. (2019). Metascape provides a biologist-oriented resource for the analysis of systems-level datasets. *Nature Communications*.

[31] Salton C. J., Chuang M. L., O’Donnell C. J. (2002). Gender differences and normal left ventricular anatomy in an adult population free of hypertension. *Journal of the American College of Cardiology*.

[32] Shannon P., Markiel A., Ozier O. (2003). Cytoscape: a software environment for integrated models of biomolecular interaction networks. *Genome Research*.

[33] Bader G. D., Hogue C. W. (2003). An automated method for finding molecular complexes in large protein interaction networks. *BMC Bioinformatics*.

[34] Song M., Zhang X., Gao Y. (2022). RNA sequencing reveals the emerging role of bronchoalveolar lavage fluid exosome lncRNAs in acute lung injury. *PeerJ*.

[35] Do-Umehara H. C., Chen C., Urich D. (2013). Suppression of inflammation and acute lung injury by Miz1 via repression of C/EBP-*δ*. *Nature Immunology*.

[36] de Souza Xavier Costa N., Ribeiro Junior G., Dos Santos Alemany A. A. (2017). Early and late pulmonary effects of nebulized LPS in mice: an acute lung injury model. *PLoS One*.

[37] Pouzol L., Sassi A., Baumlin N. (2021). CXCR7 antagonism reduces acute lung injury pathogenesis. *Frontiers in Pharmacology*.

[38] Ritchie M. E., Phipson B., Wu D. (2015). Limma powers differential expression analyses for RNA-sequencing and microarray studies. *Nucleic Acids Research*.

[39] Lockhart S. M., O’Rahilly S. (2021). Colchicine-an old dog with new tricks. *Nature Metabolism*.

[40] Lazaros G., Imazio M., Brucato A. (2018). The role of colchicine in pericardial syndromes. *Current Pharmaceutical Design*.

[41] Deftereos S., Giannopoulos G., Angelidis C. (2015). Anti-inflammatory treatment with colchicine in acute myocardial infarction: a pilot study. *Circulation*.

[42] Hennessy T., Soh L., Bowman M. (2019). The Low Dose Colchicine after Myocardial Infarction (LoDoCo-MI) study: a pilot randomized placebo controlled trial of colchicine following acute myocardial infarction. *American Heart Journal*.

[43] Aral C. A., Aral K., Yay A., Ozcoban O., Berdeli A., Saraymen R. (2018). Effects of colchicine on gingival inflammation, apoptosis, and alveolar bone loss in experimental periodontitis. *Journal of Periodontology*.

[44] Lee F. Y., Lu H. I., Zhen Y. Y. (2013). Benefit of combined therapy with nicorandil and colchicine in preventing monocrotaline-induced rat pulmonary arterial hypertension. *European Journal of Pharmaceutical Sciences*.

[45] Grommes J., Soehnlein O. (2011). Contribution of neutrophils to acute lung injury. *Molecular Medicine*.

[46] Ishii T., Doi K., Okamoto K. (2010). Neutrophil elastase contributes to acute lung injury induced by bilateral nephrectomy. *American Journal Of Pathology*.

[47] Yamada M., Kubo H., Kobayashi S. (2011). The increase in surface CXCR4 expression on lung extravascular neutrophils and its effects on neutrophils during endotoxin-induced lung injury. *Cellular and Molecular Immunology*.

[48] Li F., Xu X., Geng J., Wan X., Dai H. (2020). The autocrine CXCR4/CXCL12 axis contributes to lung fibrosis through modulation of lung fibroblast activity. *Experimental and Therapeutic Medicine*.

[49] Busillo J. M., Benovic J. L. (2007). Regulation of CXCR4 signaling. *Biochimica et Biophysica Acta (BBA)- Biomembranes*.

[50] Deftereos S., Giannopoulos G., Papoutsidakis N. (2013). Colchicine and the heart: pushing the envelope. *Journal of the American College of Cardiology*.

