# Supplementary material for: Network Pharmacology Analysis of the Therapeutic Potential of Colchicine in Acute Lung Injury
Source: Int J Clin Pract. 2024 Feb 6;2024:9940182. doi: 10.1155/2024/9940182 (PMC10864054; doi:10.1155/2024/9940182)
Supplement: Supplementary Materials — Supplementary Table 1. The expression fold change and the P values of the 15 differentially expressed genes. [file 9940182.f1.docx]

**Supplement Table 1.** The expression fold change and the P values of the 15 differentially expressed genes.

| Gene symbol | Log_2_ fold‐change | P |
| --- | --- | --- |
| CDKN1A | -1.3092 | 0.000986 |
| TM9SF4 | -1.06709 | 0.001329 |
| CDKN1C | -1.65396 | 0.003953 |
| BTNL8 | -1.47876 | 0.009589 |
| TREM1 | -1.08609 | 0.013002 |
| UPB1 | 1.027929 | 0.014491 |
| HLA | -1.4351 | 0.026972 |
| HOPX | 1.149601 | 0.031109 |
| CYBRD1 | 1.180317 | 0.033784 |
| GNAZ | -1.03549 | 0.037679 |
| PDE4B | -1.00303 | 0.040714 |
| MAP3K7CL | -1.08664 | 0.041902 |
| CAMK1 | -1.02364 | 0.047099 |
| DUSP6 | -1.04958 | 0.04777 |
| HIST1H4H | -1.03127 | 0.049362 |
